# The effect of adipose derived stromal cells on oxidative stress level, lung emphysema and white blood cells of guinea pigs model of chronic obstructive pulmonary disease

**DOI:** 10.1186/2008-2231-22-26

**Published:** 2014-02-04

**Authors:** Ahmad Ghorbani, Azadeh Feizpour, Milad Hashemzahi, Lila Gholami, Mahmoud Hosseini, Mohammad Soukhtanloo, Farzaneh Vafaee Bagheri, Esmaeil Khodaei, Nema Mohammadian Roshan, Mohammad Hossein Boskabady

**Affiliations:** 1 Neurogenic Inflammation Research Centre and Department of Physiology, School of Medicine, Mashhad University of Medical Sciences, Mashhad 9177948564, Iran; 2 Pharmacological Research Center of Medicinal Plants, School of Medicine, Mashhad University of Medical Sciences, Mashhad, Iran; 3 Neurocognitive Research Center and Department of Physiology, School of Medicine, Mashhad University of Medical Sciences, Mashhad, Iran; 4 Department of Clinical Biochemistry, School of Medicine, Mashhad University of Medical Sciences, Mashhad, Iran; 5 Department of Pathology, School of Medicine, Mashhad University of Medical Sciences, Mashhad, Iran

**Keywords:** COPD, Stromal cells, Malondialdehyde, Thiol, Emphysema, WBC, Guinea pigs

## Abstract

**Background:**

Chronic obstructive pulmonary disease (COPD) is a worldwide epidemic disease and a major cause of death and disability. The present study aimed to elucidate pharmacological effects of adipose derived stromal cells (ASCs) on pathological and biochemical factors in a guinea pig model of COPD. Guinea pigs were randomized into 5 groups including: Control, COPD, COPD + intratracheal delivery of PBS as a vehicle (COPD-PBS), COPD + intratracheal delivery of ASCs (COPD-ITASC) and COPD + intravenous injection of ASCs (COPD-IVASC). COPD was induced by exposing animals to cigarette smoke for 3 months. Cell therapy was performed immediately after the end of animal exposure to cigarette smoke and 14 days after that, white blood cells, oxidative stress indices and pathological changes of the lung were measured.

**Results:**

Compared with control group, emphysema was clearly observed in the COPD and COPD-PBS groups (p < 0.001). Lung histopathologic changes of COPD-ITASC and COPD-IVASC groups showed non-significant improvement compared to COPD-PBS group. The COPD-ITASC group showed a significant increase in total WBC compared to COPD-PBS group but there was not a significant increase in this regard in COPD-IVASC group. The differential WBC showed no significant change in number of different types of leukocytes. The serum level of malondialdehyde (MDA) significantly decreased but thiol groups of broncho-alveolar lavage fluid (BALF) increased in both cell treated groups (p < 0.05 for all cases). Weight of animals decreased during smoke exposure and improved after PBS or cell therapy. However, no significant change was observed between the groups receiving PBS and the ones receiving ASCs.

**Conclusion:**

Cell therapy with ASCs can help in reducing oxidative damage during smoking which may collectively hold promise in attenuation of the severity of COPD although the lung structural changes couldn’t be ameliorated with these pharmacological therapeutic methods.

## Background

Chronic obstructive pulmonary disease (COPD) is a progressive lung disease. Its three most common pathological changes are chronic bronchitis, chronic obstructive bronchiolitis and emphysematous destruction of the lung parenchyma [1]. Emphysema, the main histopathologic feature of COPD, leads to a decrease in elastic recoil and therefore continuation of decrease in small airway patency during expiration causing a not fully reversible airflow limitation and increase in the lung residual volume [2]. This airway obstruction may contribute to respiratory failure and weight loss leading to cachexia [3] and eventually death. Thus, finding a fundamental and curative approach for this disease is of vital importance.

The other characteristic feature of COPD, as well as other respiratory diseases, is the oxidant-antioxidant imbalances. This imbalance is reported to be developed by the increased lipid peroxidation which is known as an index of oxidative stress and a reduction of antioxidant capacity. Decrease in plasma protein sulfhydryl concentrations in COPD patients is well documented [4]. There are also other evidences suggesting the role of reactive oxygen species in lung inflammation of COPD patients which can be a direct effect or through lipid peroxidation products [5]. Therefore, increasing the antioxidant capacity is one of the main concerns in the pharmacological treatment of COPD.

For treatment of such respiratory diseases as asthma, bronchodilating agents are helpful but the airflow limitation and accelerated loss of lung function caused by COPD cannot be reversed by this type of treatments [4,6]. The existing treatments for COPD only change the symptoms, quality of life and exacerbation frequency, while a fundamental or effective therapy is yet to be achieved. Recent advances in regenerative medicine and cell therapy have led to successful attempts to restore damaged tissues. Therefore, cell-based therapy might be a more promising therapeutic option for COPD.

Adipose tissue derived stromal cells (ASCs) and mesenchymal stem cells (MSCs), derived from ASCs or bone marrow, are readily available sources of stromal/stem cells required for cell therapy [5-10]. Many researchers such as Schweitzer et al. [5], Ahmed et al. [6], Gupta et al. [7], Baber et al. [8], Shigemura et al. [9] and Ishiazwa et al. [10] have contributed to cell therapy of lung diseases by local or systemic administration of ASCs/MSCs to the animal model of different lung injuries induced by elastase, monocrotaline or endotoxin and have observed promising pharmacological effects.

The MSCs are reported to interfere with oxidative stress and induce lung parenchymal regeneration [5,11]. Mesenchymal stem cells can ameliorate the pulmonary damages by two mechanisms. One is protecting the vascular bed endothelial cells from apoptosis by paracrine effects of several growth factors such as Hepatocyte growth factor (HGF) and Vascular endothelial growth factor (VEGF). The other mechanism is acquiring the phenotype or markers of airway or alveolar epithelial cells and vascular endothelial cells. In addition, it has been shown that transplantation of ASCs into elastase-treated emphysema models augments alveolar and vascular regeneration by enhancement of epithelial cell proliferation, inhibition of alveolar cell apoptosis and promotion of angiogenesis in lung vasculature [9,12]. Lung emphysematous destruction induced by COPD is well studied by different studies. However, MSCs ameliorative effect on COPD in guinea pigs, the main model of respiratory diseases, induced by cigarette smoking, the main cause of COPD in human was not investigated. On the other hand, some researches documented a pharmacological protective effect on the epithelial cells during oxidative damage by involvement of such cytokines as insulin-like growth factor (IGF) [7,8], HGF [9], and IL-6 [13] which raise the question if the MSCs may exert their therapeutic effect by an antioxidant function through secreting cytokines. There are few studies on the topic of antioxidant function of stem cells such as the one documenting that MSC protection of the kidneys against ischemia/reperfusion injury may be at least in part due to their antioxidant effects [14]. Another study also confirms an antioxidant along with a neuroprotective function for MSCs which have been previously approved in an *in vitro* study on neuroblastoma cells exposed to an oxidative stress [15]. However, such studies aren’t formerly performed on the oxidative damage induced in the lungs of COPD model guinea pigs.

Considering the pathological changes occurring during COPD, at least three targets exist for intervention including inflammation, emphysematous destruction, and oxidative stress [5,14,15] among which the two latter ones are the focus of this study.

As far as we know, there is no study to investigate the ameliorative effect of ASCs on cigarette smoking-induced lung injury in guinea pigs, the main animal model of COPD. Therefore, the present study aimed to elucidate the pharmacological effects of local and systemic injection of ASCs on oxidative factors, lung pathology and white blood cells in cigarette smoke induced COPD in guinea pigs.

## Methods

### Animals and groups

Thirty one guinea pigs (600–800 g) were kept in a temperature controlled room while a 12-h on/12-h off light cycle was maintained. All animal experiments were carried out according to the ethical guidelines of the animal care of the Mashhad University of Medical Sciences. They were categorized to 5 groups as follows:

a) Control group, the animals were exposed to ambient air rather than smoke (n = 6).

b) COPD group, the animals were exposed to cigarette smoke for 3 consecutive months (n = 9).

c) COPD + PBS, the animals were exposed to cigarette smoke and then received PBS as vehicle via intratracheal injection (n = 5).

d) COPD + intratracheal delivery of ASCs (COPD-ITASC), the animals were exposed to cigarette smoke and then received 10^6^ ASCs via intratracheal injection (n = 6).

e) COPD + intravenous injection of ASCs (COPD-IVASC): the animals were exposed to cigarette smoke and then received 10^6^ ASCs via intravenous injection (n = 5).

Biochemical assays and lung pathological examination were done 14 days after treatment with cell or vehicle.

### Exposure of animals to cigarette smoke

Exposure of guinea pigs to cigarette smoke was performed according to the method designed by Boskabady and Kiani with some modifications [16]. Briefly, the animals were placed in a special box which was divided into two parts: one held the body of the animal and the other one held its head (dimensions: 15×12×7 cm). Twenty millilitre puffs of cigarette smoke were drawn out of the cigarettes using a syringe and then exhausted at a rate of two puffs per minute into the animals’ head chamber (every 30 seconds, one puff of cigarette smoke was dragged into the head chamber). Exposure of animals to each cigarette lasted for 8–9 minutes, with a 10 minutes resting period between two cigarettes. The animals were exposed initially to one cigarette per day and gradually increasing to a maximum of 5 cigarettes per day over a period of 20 days. In short, the animals were exposed to cigarette smoke (Magna: Nicotine = 5, tar = 6) for totally 3 consecutive months, 5 days per week, and 5 cigarettes per day (the cigarettes’ filters weren’t removed).

### Preparation of stromal cells

Adipose tissues were obtained from healthy guinea pigs weighing 600–800 g. They were anesthetized by intra peritoneal injection of ketamine (150 mg/kg) and xylazine (6 mg/kg). Subcutaneous inguinal fat deposits were resected and under laminar hood, the fat tissue was minced into 1–2 mm pieces by means of a sterile scalpel [17]. The tissue pieces were incubated at 37°C for 60 minutes in PBS containing 2 mg/ml collagenase meanwhile being shaken (60 cycles/min) [18,19]. After centrifugation (300 g for 8 minutes), the floated lipid layer was discarded and the stroma-vascular fraction was collected, washed and re-suspended in DMEM medium supplemented with 10% FBS, 100 units/ml penicillin and 100 μg/ml streptomycin [19,20]. The cells were seeded into tissue culture flask and passaged when 60–80% confluent and used at passages three to six.

### Differentiation of stromal cell

For differentiation to adipocyte, the stromal cells were seeded in 12-well culture plate and then incubated in DMEM supplemented with 3% FBS, 66 μM biotin, 250 μM IBMX, 1 μM dexamethasone, 34 μM d-panthothenate, 5 μM indomethacin and 0.2 μM insulin. The cells were maintained in differentiation medium for 3 days and then exposed to the adipocyte maintenance medium consisting of DMEM, supplemented with 3% FBS, 66 μM biotin, 1 μM dexamethasone, 34 μM d-panthothenate and 0.2 μM insulin. After additional 9 days of incubation, adipogenesis was confirmed by Oil Red O which stains intracellular triglyceride droplets. For staining, the cells were fixed with 10% formalin and then incubated with Oil Red O solution. Thereafter, the cells were washed three times with distilled water and photographed using inverted microscope [19].

For osteocyte differentiation, the stromal cells were incubated in DMEM supplemented with 10% FBS, 10 μg/ml ascorbic acid, 5 mM β-glycerol phosphate and 0.1 μM dexamethasone. The cells were maintained in the differentiation medium for two weeks and the culture medium was replaced every 3 days [21]. For Alizarin red staining, the cells were fixed with 10% formalin and then incubated with 2% Alizarin red solution. Thereafter, the cells were washed three times with distilled water and photographed using inverted microscope [22].

### Stromal cell labeling and tracing

The cells were harvested, suspended in PBS and incubated with 2 μM cell tracker CM DiI (Invitrogen) for 5 min in 37°C and 15 min in 4°C in the absence of light. After 20 min of staining, it was centrifuged at 1500 for 5 min, washed with PBS, centrifuged again and suspended in 0.3 ml PBS. It was finally dragged to a 27 gauge insulin syringe and was prepared for injection to the animals’ jugular vein or trachea. After 2 weeks of ASCs administration (either intratracheal or intravenous), the animals were euthanized and 4 μm sections were provided from different regions of their lung. Existence of the CM-DiI labeled stromal cells was detected under fluorescent microscope.

### Intratracheal and systemic delivery of stromal cells

The guinea pigs of COPD-ITASC group were anesthetized and after exposing the trachea, 0.3 ml PBS containing 10^6^ cells was injected under direct vision to the trachea using a 27 gauge insulin syringe. Viability of injected cells was found to be more than 90% as assessed with trypan blue staining.

In COPD-IVASC group, the animals were anesthetized and the jugular vein was exposed. Then, a volume of 0.3 ml PBS containing 10^6^ cells was injected into the vein using a 27 gauge insulin syringe.

### Total WBC and differential WBC measurement

Total WBC was counted in duplicate in a hemocytometer (in a Burker chamber) in blood stained with Turk solution (1:10 dilution, consisted of 1 ml of glacial acetic acid, 1 ml of gentiac vialet solution 1% and 100 ml distilled). Differential WBC was determined in blood samples stained with Turk solution and Wright-Giemsa, respectively. Briefly, differential cell counts were done on thin slide, prepared with smearing blood sample, using Wright-Giemsa stain. According to staining and morphological criteria, differential cell analysis was carried out under a light microscope by counting 100 cells, and the percentage of each cell type was calculated.

### Biochemical assays

After sacrificing the guinea pigs, broncho-alveolar lavage fluid (BALF) was prepared from the lung by locating a cannula into trachea and lavage of the lungs with 2 mL of saline for 5 times (total: 10 mL). BALF was then centrifuged at 2500 *× g* for 10 min and the supernatant was collected for measurement of thiol groups’ concentration.

Total thiol groups were measured using 2,2′-dinitro-5,5′-dithiodibenzoic acid (DTNB) which reacts with the SH groups to produce a yellow colored complex with peak absorbance at 412 nm [23,24]. In summary, 1 mL Tris-EDTA buffer was added to 50 μL of BALF and sample absorbance was read at 412 nm against Tris-EDTA buffer alone (A1). Then, 20 μL of DTNB reagent (10 mM in methanol) was added to the mixture and after 15 min, the sample absorbance was again read (A2). Total thiol concentration was calculated using this equation: Total thiol (mM) = (A2-A1-blank) × 1.07/0.05 × 13.6.

Five ml blood sample was collected gently from the left ventricle and placed in a citrate containing blood collection tube not to be coagulated. The blood was centrifuged and the serum was separated and kept in −70°C for further measurement of MDA concentrations. MDA level, as an index of lipid peroxidation, was measured. MDA reacts with thiobarbituric acid (TBA) as a thiobarbituric acid reactive substance (TBARS) to produce a red colored complex which has peak absorbance at 535 nm. Two mL from reagent of TBA/trichloroacetic acid (TCA)/hydrochloric acid (HCL) was added to 1 mL of serum, and the solution was heated in a water bath for 40 minutes. After cooling, the whole solutions were centrifuged at 1000 g for 10 minutes. The absorbance was measured at 535 nm [25]. The MDA concentration was calculated as follows: C (m) = Absorbance/(1.56 × 10^**5**^).

### Lung pathology

After fixation of the lung specimen in formalin and staining with H&E, the tissue sections were evaluated under a light microscope by a pathologist. The pathologic changes in the lung of COPD, COPD-PBS, COPD-ITASC and COPD-IVASC groups were evaluated according to the intensity of emphysema. Three intensities of parenchymal destruction were considered for scoring the emphysema observed in the lungs. Scoring of pathological changes was performed as previously described [16]. For this purpose, the percent of the area containing mild, moderate or severe emphysema was multiplied by the number 1, 2 or 3, respectively and the total score for each section was calculated.

### Animals weight measurement

The weight of the animals of all groups was measured at the beginning of the study (week 0) and after 3 months (at the end of exposure period); but in COPD-PBS, COPD-ITASC and COPD-IVASC groups, the weight of animals was also measured after a further 2 weeks (at the end of treatment with PBS or ASC cells).

### Statistical analysis

All the data were quoted as mean ± SEM and compared by means of Instat software. The data of COPD animals were compared to the control ones using unpaired “t” test. The same test was used for the comparison between PBS and either COPD + ITASC or COPD + IVASC as well as between COPD + PBS and COPD. In addition, the comparison between the weights of animals at three time points during the procedure was performed using ANOVA. Significance was accepted at p < 0.05.

## Results

### Stromal cell characterization

**Figure 1:**
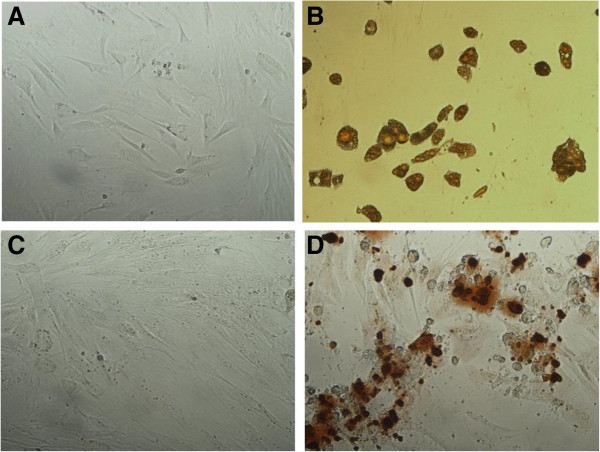
**Differentiation of adipose derived stromal cells to adipocyte and osteocyte lineages.** The stromal cells were cultured in adipogenic or osteogenic differentiating media for 12 and 14 days, respectively. **A**: Oil Red O staining of cells cultured in control media; **B**: Oil Red O staining of cultured cells in adipogenic differentiating media; **C**: Alizarin Red staining of cells cultured in control media; **D**: Alizarin Red staining of cells cultured in osteogenic differentiating media. Magnification × 100.

Figure 1A and C shows the morphology of stromal cells isolated from adipose tissue of guinea pig. The cells were adherent and showed significant expansion in the cultures (Figure 1A). After passage 3, they had a spindled, fibroblast appearance in culture that is consistent with MSCs morphology.

To determine whether the cells have pluripotent capacity, before intratracheal or intravenous injection, a number of them were cultured in differentiating media specific for adipocyte and osteocyte. Oil Red O staining showed the accumulated triglyceride droplets in the cells cytoplasm which confirms adipogenic differentiation capacity of the isolated stromal cells (Figure 1B). In addition, Alizarin Red staining revealed extracellular matrix mineralization which confirms osteogenic differentiation capacity of the stromal cells (Figure 1D).

### Stromal cell detection in the lung

**Figure 2:**
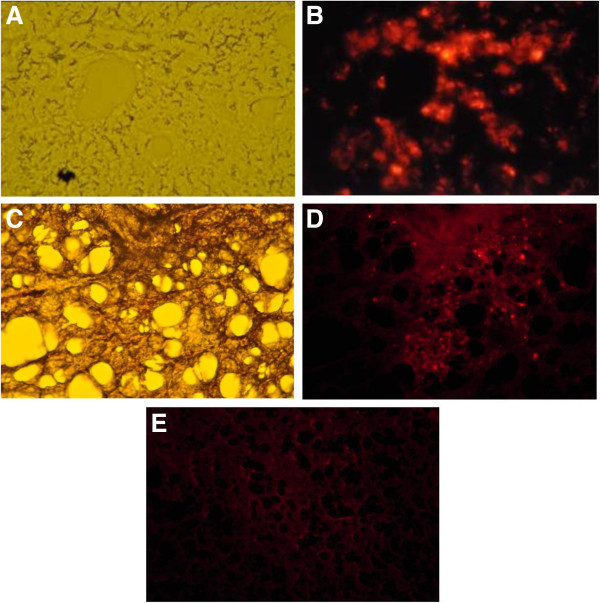
**Fluorescence microscopic photographs of lungs after intratracheal delivery of CMDiI-labeled stromal cells. A**: Phase-contrast microscopic image of lung from normal animal harvested 2 days after injection of labeled stromal cells into trachea (Magnification × 200); **B**: The same field under fluorescent microscope. **C**: Phase-contrast microscopic image of formalin fixed lung from COPD animal harvested 14 days after injection of labeled stromal cells into trachea (Magnification × 100); **D**: The same field under fluorescent microscope; **E**: Fluorescence microscopic image of lung from COPD animal harvested 14 days after injection of PBS as vehicle (Magnification × 100).

To detect stromal cells in airway structures, the CM-DiI labeled cells were injected to trachea. Figure 2 shows fluorescence microscopy of stromal cells after 2 or 14 days of intratracheal delivery. Two days after intratracheal injection, microscopic image of lung from normal guinea pig (guinea pig experiencing no treatment) confirmed the delivery of stromal cells into both large and small airway structures (Figure 2A and B). The CM-DiI-labeled stromal cells were also detected in the lung from COPD guinea pig 14 days after intratracheal administration (Figure 2C and D). There was no fluorescence signal in the lung harvested from COPD animal 14 days after injection of PBS as vehicle (Figure 2E).

**Figure 3:**
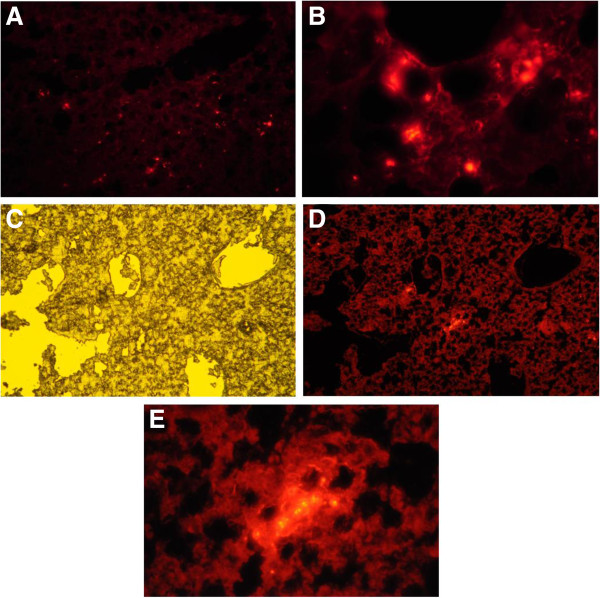
**Fluorescence microscopic photographs of lungs after systemic delivery of CMDiI-labeled stromal cells. A** and **B**: Lung homing of labeled stromal cells two days after injection of cells into jugular vein of normal guinea pig (Magnification: A = ×100, B = ×400); **C**: Phase-contrast microscopic image of formalin fixed lung from COPD animal harvested 14 days after injection of labeled stromal cells into jugular vein (Magnification × 100); **D**: The same field under fluorescent microscope (Magnification × 100); **E**: The same field under fluorescent microscope with magnification of × 400.

To ensure pulmonary delivery of stromal cells after intravenous injection, fluorescence microscopy was also done on lung sections two days after cell injection. The labeled cells were detected in the lung alongside resident cells in airways and alveolar structures (Figure 3A and B). In separate homing experiments, the labeled cells were administered systemically via intravenous injection to a COPD guinea pig. Fourteen days after administration of the cells, fluorescence signals of labeled cells were still detected in the lung (Figure 3C, D and E).

### Lung histopathological results

**Figure 4:**
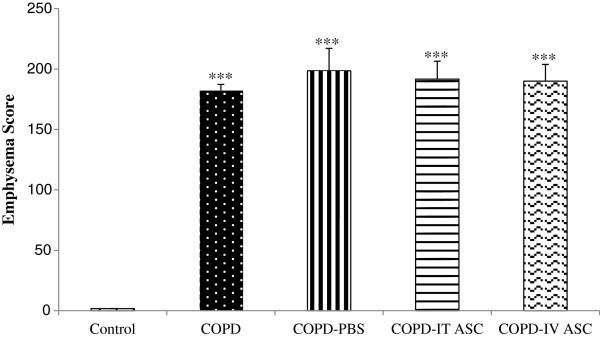
**Effect of adipose derived stromal cells (ASCs) therapy on pathological changes of the lung according to the scoring method mentioned in the text.** Data are shown as mean ± SEM. ***P < 0.001 as compared with control group. The animals in COPD-PBS, COPD-ITASC and COPD-IVASC groups were exposed to cigarette smoke for 3 months and then received PBS, intratracheal injection of ASCs and intravenous injection of ASCs, respectively. For scoring, the percent of the area containing mild, moderate or severe emphysema was multiplied by the number 1, 2 or 3, respectively.

Three consecutive months of exposing animals to the cigarette smoke caused a considerable destruction of alveolar walls and consequently widespread emphysema as H&E stained sections show. The same changes were observed in the COPD-PBS group and there was no significant difference between the emphysema score in COPD-PBS and COPD groups. The change in emphysema score of COPD-ITASC and COPD-IVASC animals was non-significant compared to the COPD-PBS group (Figure 4).

### Biochemical results

**Figure 5:**
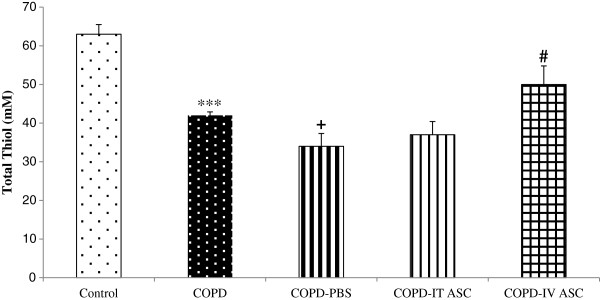
**Thiol total concentration in broncho-alveolar lavage fluid in different groups.** Data are shown as mean ± SEM. ***P < 0.001 as compared with control group. ^#^*P <* 0.05 as compared with COPD-PBS group. ^+^*P <* 0.05 as compared with COPD group. The animals in COPD-PBS, COPD-ITASC and COPD-IVASC groups were exposed to cigarette smoke for 3 months and then received PBS, intratracheal injection of ASCs and intravenous injection of ASCs, respectively.

BALF thiol concentration had a significant decrease in COPD compared with control group (*P <* 0.001). There was also a significant reduction in this factor in COPD-PBS group compared to the COPD animals (*P <* 0.05). The thiol concentration increased in both treated groups with stromal cells, but it was only significant in COPD-IVASC compared to COPD-PBS group (*P <* 0.05), (Figure 5).

**Figure 6:**
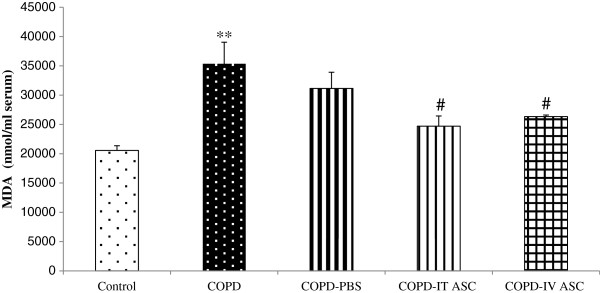
**Serum concentration of malondialdehyde (MDA) in different groups.** Data are shown as mean ± SEM. **P < 0.01 as compared with control; ^#^*P <* 0.05 as compared with COPD-PBS. The animals in COPD-PBS, COPD-ITASC and COPD-IVASC groups were exposed to cigarette smoke for 3 months and then received PBS, intratracheal injection of ASCs and intravenous injection of ASCs, respectively.

Concentration of MDA had a significant increase in the COPD group compared with the control animals (*P <* 0.01) which was not significantly different with COPD-PBS groups (Figure 6). The concentration of MDA in both treated groups with the stromal cells showed a significant reduction compared to the COPD-PBS animals (*P <* 0.05).

### Total and differential WBC counts

**Figure 7:**
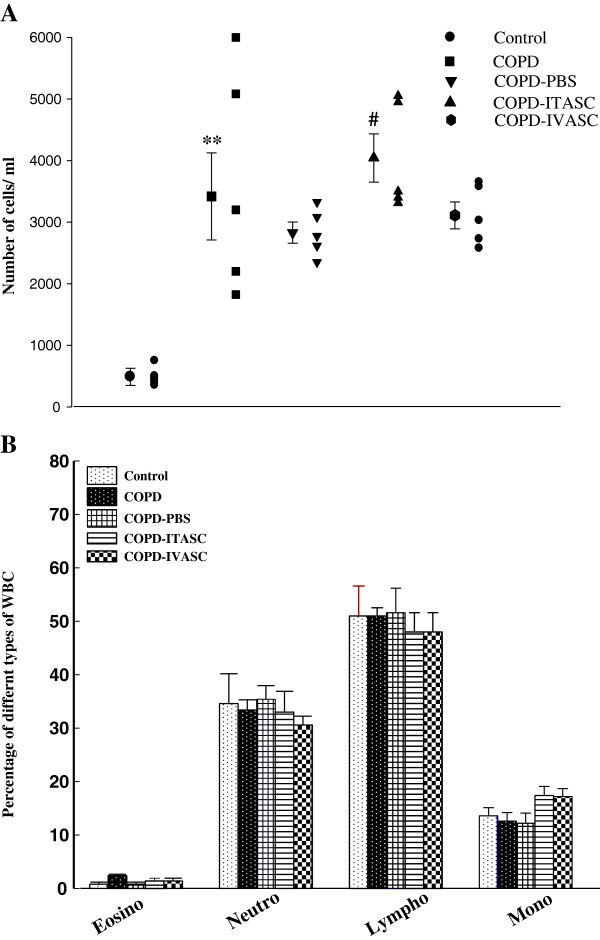
**Effects of adipose derived stromal cells therapy on white blood cell counts in blood of different groups.** Effects of adipose derived stromal cells (ASCs) on total white blood cell (WBC), **(A)** and its effect on differential WBC **(B)** counts. Data are shown as mean ± SEM. The animals in COPD-PBS, COPD-ITASC and COPD-IVASC groups were exposed to cigarette smoke for 3 months and then received PBS, intratracheal injection of ASCs and intravenous injection of ASCs, respectively.

There was a significant increase in total WBC in the COPD compared to the control group (*P <* 0.01). No significant difference was observed between the total WBC counts in COPD-PBS and COPD group (Figure 7A). There was a significant increase in this parameter in the COPD-ITASC group compared to COPD-PBS animals but no significant difference was observed between COPD-IVASC and COPD-PBS groups. There was no significant difference in the number of eosinophil, neutrophils, lymphocytes and monocytes between various groups (Figure 7B).

### Animals weight changes

**Figure 8:**
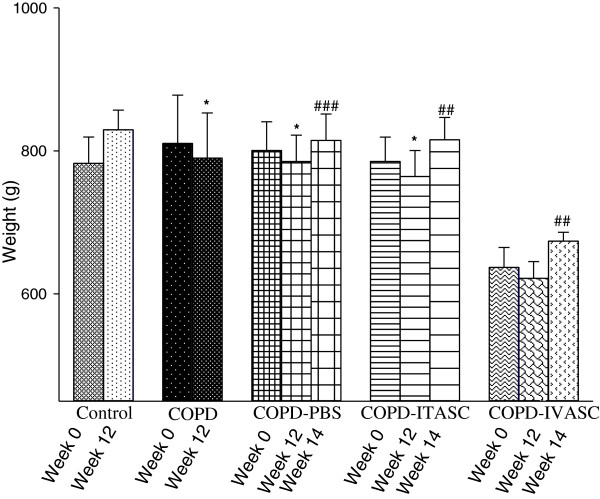
**Body weight in guinea pigs of different groups.** Data are shown as mean ± SEM values of body weight at three time points: at the first of the 3 months of inhalation (Week 0), on the last day of the 3 months of inhalation (Week 12) and 2 weeks after surgery (Week 14). *P < 0.01 as compared with week 0; ^##^*P <* 0.01 and ^###^*P <* 0.001 as compared with week 12. The animals in COPD-PBS, COPD-ITASC and COPD-IVASC groups were exposed to cigarette smoke for 3 months and then received PBS, intratracheal injection of ASCs and intravenous injection of ASCs, respectively.

There was an increase in the weight of the animals of control group after 3 months of ambient air exposure but all the other groups showed a significant reduction in this parameter after 3 months of cigarette smoking except for COPD-IVASC group in which this increase wasn’t significant (Figure 8). After the surgery and injection of stromal cells or PBS, all the three groups showed a significant increase in animals’ weight during the two weeks before being euthanized.

## Discussion

In many studies pertaining to asthma and COPD, the most widespread small animal species used are the mice or rats while guinea pigs are the most susceptible species and a significant airspace enlargement happens in their lungs after a few months of cigarette smoke exposure [26]. Besides, the most considerable advantage of guinea pigs, as a respiratory disease model, its airway pharmacology and physiological processes of these animals are similar to human such as the airway autonomic system, allergic reaction and responses to agonists and antagonists. The other side of the coin is that using guinea pigs in animal studies has some disadvantages too, including absence of transgenic animals, a lack of variety in guinea pig strains, and a noticeable axon reflex that is questionable whether it is present in human airways or not [27]. Therefore, despite the easier procedures and lower expenses in working with rats or mice, guinea pig was chosen as an animal model of COPD in the present study.

It is documented that the structural changes considered important in COPD are as follows: destruction of alveolar wall and existence of emphysema, alveolar space enlargement due to emphysema, increase in alveolar septum due to infiltration of leukocytes and penetration of smoke particles, elastolysis and collagen fiber deposition in alveolar septa and pulmonary vessel wall [16]. The major reasons for these changes caused by cigarette smoking are inflammatory responses, oxidative damage and changes in protease-anti protease balance. According to the results of the present study, emphysema was observed in the lungs of the smoke-exposed group of animals, so that the adjacent alveolar spaces were joined to each other due to the parenchymal destructions and air space enlargements were clearly observed. This is consistent with other studies demonstrating physiological and morphological changes induced by cigarette smoke in lung of guinea pigs [16,27,28]. In the present study, treatment of smoke exposed animals by stromal cells showed no significant improvement in the intensity of the smoke-induced emphysema. As all the procedure of familiarizing animals to the new habitat, smoke exposure and cell administrations took several months, it was assumed that higher age of the animals at the end of the study might be interfering with cell therapy. Therefore, the inconvenient histopathology result might be due to the lack of regenerative mechanisms or decreased numbers of progenitor cells in the lung parenchyma or a stromal vascular fraction of the older subjects [10]. Kasper et al. also worked on MSC properties in bone marrow; they documented that not only these cells’ potential regeneration decreases with age but also their migration capability and number in bone marrow decreases as an individual grows older [29]. Furthermore, the number of systemically or locally injected cells varies from 1 × 10^6^ to 5 × 10^7^ in different studies on the effect of MSCs on lung injuries [9,10,30]. Accordingly, another reason of the observed result in this part of our study might be due to the inevitable low number of injected cells. The number of stromal cells was intentionally chosen to be low in this experiment because in a study, carried out by Lee et al., it was demonstrated that intravenous injection of high number of MSC into mice contributed in embolism in the lung which may be fatal [31]. Consistently, Furlani et al. documented that some of their animals died of pulmonary embolism after abdominal aorta injection of high and low doses of MSCs (1 × 10^6^ MSCs or 0.2 × 10^6^), while no death was observed in the present study [32]. In addition, one of the most probable reasons for the absence of improving pharmacological effects of cell therapy on structural changes of the lung is that these changes take place during a long period of development of COPD which is well known to be rarely reversible. For this reason, in further studies, the effect of cell therapy is recommended to be examined during different stages of development of COPD not at the end of the disease development.

The results of this study also showed increased total WBC in the blood of smoke exposed animal and COPD-PBS group. However, cell therapy did not affect changes in blood total WBC. The probable reasons for the absence of improving effects of cell therapy on this parameter could also be due to taking place of this change during a long period of development of COPD which is difficult to be affected by this method of therapy. However, further studies regarding the effect of cell therapy during different stages of development of COPD could be of great value. The ranges of differential WBC counts reported in this study may seem inconsistent with normal values. However, the ranges for lymphocyte, neutrophil and eosinophil number are 30-80%, 20-60% and 0-7% respectively in guinea pig which is different from human [33]. Our previous studies also showed similar range of differential WBC counts in guinea pigs [34,35].

In the present study, the cells injected into the trachea or the jugular vein of the animals were traced to confirm that they have reached the injured lung. As described before, the labeled stromal cells were detectable in the tissue even 2 weeks after cell therapy. It is consistent with another study reporting the persistence of MSCs 21 days following their injection into the trachea [8]. Schweitzer et al. also documented that systemically delivered adipose stem cells were detectable in the parenchyma and large airways of the lungs up to 21 days after injection [5]. It seems that two weeks recovery, given to the animals after administration of the cells, is a proper time for the stromal cells to exert their impact either by regeneration in the damaged tissue or by paracrine effects.

The results of the present study showed that the oxidative stress was increased in the smoke exposed group both in their BALF and serum measured by an increase in the concentration of MDA in serum and a decrease in the thiol groups in BALF. It is well documented that cigarette smoke contains stable compounds that undergo redox-cycle to form reactive oxygen species such as superoxide radicals, hydrogen peroxide, hydroxyl radicals etc. [36]. Our results are in accordance with the study carried out by Qamar et al., who reported the debility in antioxidant defenses after intratracheal instillation of cigarette smoke extract in rats [36]. Since there was no significant difference in the MDA and thiol concentration between COPD and COPD-PBS groups, it can be concluded that the PBS injection had no pharmacological effect on the parameter under investigation. BALF thiol concentration of the treated groups with intratracheal and intravenous administration of stromal cells increased significantly in COPD-IVASC group. However, both treated groups showed significant decrease in serum MDA concentration. These findings confirmed the ameliorative effect of stromal cells on the oxidative damage caused by cigarette smoke. Consistently, the antioxidant effect of mesenchymal stem cells within the stromal-vascular fraction of subcutaneous adipose tissue has been previously shown by Kim et al. documenting that these cells have potent antioxidant activity and protect HDFs from oxidative injury by decreasing apoptotic cells [11].

Considering the results, although the animals were kept in boxes with low possibility of movement and high probability of gaining weight during three months, being exposed to cigarette smoke during these months caused a considerable decrease in body weight of almost all the animals. Complied with these results, Schweitzer et al. documented that patients affected by emphysema often exhibit progressive respiratory symptoms and loss of lung function culminating in respiratory failure and systemic weight loss [5]. There are plenty of other studies demonstrating the effect of cigarette smoking on body weight loss [37,38]. In the present study, after finishing the three months of smoke exposure, the surgery and administration of the cells or PBS to the animals led to a significant increase in body weight. As Schweitzer et al. documented, therapeutic effects of adipose stem cells aren’t restricted to the lung but also contains restoring the weight loss sustained by guinea pigs during cigarette smoke exposure [5]. However, the weight gain after the surgery, in the present study, can’t be associated with the treatment with stromal cells because this improvement was observed in the PBS receiving groups too. Therefore, it can be concluded that smoke cessation and lower mobility of the animals due to the surgery might be the initial source of gaining weight.

In the present study, the applied method of exposing the animals to cigarette smoke resulted in exposure of all the animals to the same concentration of cigarette smoke as follows; every 30 second a twenty ml puff of smoke was dragged into the head chamber using a 20 ml syringes and duration of exposure of the animals to each cigarette was about 8 minutes with the same duration of rest between each two successive cigarettes. This protocol was maintained during the whole procedure and the smoke concentration in the head chamber was maintained the same for all the groups. This obviously leads to the similar levels of plasma nicotine and its metabolite as well as similar levels of plasma carboxyhemoglobin. Therefore, the variations observed in the results of different groups are just due to the variations in treatment protocol between them.

## Conclusions

In conclusion, both intratracheal and intravenous cell therapy lead to pharmacological effect on reduction of oxidative damage and restoring the weight loss during smoking which may collectively hold promise in attenuation of the severity of the disease in the patients experiencing COPD although the lung structural changes couldn’t be ameliorated with these therapeutic methods. However, more investigations are needed to further assess the pharmacological effects of adipose derived stromal cells on the microscopic structure of the damaged lung induced by cigarette smoke.

## Competing interest

The authors declare that they have no competing interest.

## Authors’ contributions

AG: help in study design, supervision of experiments, statistical analysis and preparation of manuscript, AF: performance of experiment, help in statistical analysis and manuscript preparation, MH: help in performance of experiment, LG: help in performance of experiment, MH: help in study design and supervision of experiments, MS: Bichemichal analysis, FVB: help in performance of experiment, EK: help in performance of experiment, NMR: Patholofical evaluations, MHB: study design, supervision of experiments, help in statistical analysis and preparation of manuscript. All authors read and approved the final manuscript.

## References

[B1] Pauwels RA Buist AS Ma P Jenkins CR Hurd SS Global strategy for the diagnosis, management, and prevention of chronic obstructive pulmonary disease: National Heart, Lung, and Blood Institute and World Health Organization Global Initiative for Chronic Obstructive Lung Disease (GOLD): executive summary Resp Care 2001 46 798 825 11463370

[B2] Saetta M Ghezzo H Kim W King M Angus G Wang N Loss of alveolar attachments in smokers. A morphometric correlate of lung function impairment Am Rev Respir Dis 1985 132 894 900 4051324 10.1164/arrd.1985.132.4.894

[B3] Jeffery PK Structural and inflammatory changes in COPD: a comparison with asthma Thorax 1998 53 129 136 10.1136/thx.53.2.129 9624299 PMC1758710

[B4] Celli B MacNee W Agusti A Anzueto A Berg B Buist A Standards for the diagnosis and treatment of patients with COPD: a summary of the ATS/ERS position paper Eur Respir J 2004 23 932 946 10.1183/09031936.04.00014304 15219010

[B5] Schweitzer KS Johnstone BH Garrison J Rush NI Cooper S Traktuev DO Adipose stem cell treatment in mice attenuates lung and systemic injury induced by cigarette smoking Am J Respir Crit Care Med 2011 183 215 225 10.1164/rccm.201001-0126OC 20709815 PMC3040390

[B6] Gladysheva ES Malhotra A Owens RL Influencing the decline of lung function in COPD: use of pharmacotherapy Int J COPD 2010 5 153 164 10.2147/copd.s4577 PMC2898088 20631815

[B7] Gupta N Su X Popov B Lee JW Serikov V Matthay MA Intrapulmonary delivery of bone marrow-derived mesenchymal stem cells improves survival and attenuates endotoxin-induced acute lung injury in mice J Immunol 2007 179 1855 1863 17641052 10.4049/jimmunol.179.3.1855

[B8] Baber SR Deng W Master RG Bunnell BA Taylor BK Murthy SN Intratracheal mesenchymal stem cell administration attenuates monocrotaline-induced pulmonary hypertension and endothelial dysfunction Am J Physiol-Heart C 2007 292 1120 10.1152/ajpheart.00173.2006 16980338

[B9] Shigemura N Okumura M Mizuno S Imanishi Y Nakamura T Sawa Y Autologous transplantation of adipose tissue‒derived stromal cells ameliorates pulmonary emphysema Am J Transplant 2006 6 2592 2600 10.1111/j.1600-6143.2006.01522.x 17049053

[B10] Ishizawa K Kubo H Yamada M Kobayashi S Numasaki M Ueda S Bone marrow-derived cells contribute to lung regeneration after elastase-induced pulmonary emphysema FEBS Lett 2004 556 249 252 10.1016/S0014-5793(03)01399-1 14706858

[B11] Kim W-S Park B-S Kim H-K Park J-S Kim K-J Choi J-S Evidence supporting antioxidant action of adipose-derived stem cells: protection of human dermal fibroblasts from oxidative stress J Dermatol Sci 2008 49 133 142 10.1016/j.jdermsci.2007.08.004 17870415

[B12] Ahmed M Katsha SO Xin H Kanehira M Sun R TNaY S Paracrine factors of multipotent stromal cells ameliorate lung injury in an elastase-induced emphysema model ASGCT 2010 19 196 203 10.1038/mt.2010.192 PMC3017437 20842104

[B13] Yamamoto C Yoneda T Yoshikawa M Fu A Tokuyama T Tsukaguchi K Airway inflammation in COPD assessed by sputum levels of interleukin-8 CHEST J 1997 112 505 510 10.1378/chest.112.2.505 9266891

[B14] Drost E Skwarski K Sauleda J Soler N Roca J Agusti A Oxidative stress and airway inflammation in severe exacerbations of COPD Thorax 2005 60 293 300 10.1136/thx.2004.027946 15790984 PMC1747355

[B15] MacNee W Oxidative stress and lung inflammation in airways disease Eur J Pharmacol 2001 429 195 207 10.1016/S0014-2999(01)01320-6 11698041

[B16] Boskabady MH Kiani S The effect of exposure of guinea pig to cigarette smoke and their sensitization in tracheal responsiveness to histamine and histamine receptor (h1) blockade by chlorpheniramine Pathophysiol 2007 14 97 104 10.1016/j.pathophys.2007.06.003 17707616

[B17] Ghorbani A Varedi M Hadjzadeh MR Omrani GH Type-1 diabetes induces depot-specific alterations in adipocyte diameter and mass of adipose tissues in the rat Exp Clin Endocrinol Diabetes 2010 118 442 448 10.1055/s-0030-1247566 20198560

[B18] Ghorbani A Hadjzadeh MR Rajaei Z Zendehbad SB Effects of fenugreek seeds on adipogenesis and lipolysis in normal and diabetic rat Pak J Biol Sci 2014 17 523 528 10.3923/pjbs.2014.523.528 25911840

[B19] Ghorbani A Jalali SA Varedi M Isolation of adipose tissue mesenchymal stem cells without tissue destruction: a non-enzymatic method Tissue Cell 2014 46 54 58 10.1016/j.tice.2013.11.002 24321269

[B20] Ghorbani A Abedinzade M Comparison of *in vitro* and *in situ* methods for studying lipolysis ISRN Endocrinol 2013 2013 205385 24024037 10.1155/2013/205385 PMC3760109

[B21] Hsu LW Goto S Nakano T Chen KD Wang CC Lai CY The effect of exogenous histone H1 on rat adipose-derived stem cell proliferation, migration, and osteogenic differentiation *in vitro* J Cell Physiol 2012 227 3417 3425 10.1002/jcp.24042 22223405

[B22] Raynaud C Maleki M Lis R Ahmed B Al-Azwani I Malek J Comprehensive characterization of mesenchymal stem cells from human placenta and fetal membrane and their response to osteoactivin stimulation Stem Cells Int 2012 2012 658356 22701494 10.1155/2012/658356 PMC3373208

[B23] Sadeghnia HR Yousefsani BS Rashidfar M Boroushaki MT Assadpour E Ghorbani A Protective effect of rutin on hexachlorobutadiene-induced nephrotoxicity Ren Fail 2013 35 1151 1155 10.3109/0886022X.2013.815546 23876083

[B24] Sadeghnia HR Kamkar M Assadpour E Boroushaki MT Ghorbani A Protective effect of safranal, a constituent of Crocus sativus, on quinolinic acid-induced oxidative damage in rat hippocampus Iran J Basic MedSci 2013 16 73 82 PMC3637907 23638295

[B25] Khodabandehloo F Hosseini M Rajaei Z Soukhtanloo M Farrokhi E Rezaeipour M Brain tissue oxidative damage as a possible mechanism for the deleterious effect of a chronic high dose of estradiol on learning and memory in ovariectomized rats Arq Neuro-Psiquiat 2013 71 313 319 10.1590/0004-282X20130027 23689409

[B26] Wright JL Churg A Cigarette smoke causes physiologic and morphologic changes of emphysema in the guinea pig Am J Res Crit Care Med 1990 142 1422 1428 10.1164/ajrccm/142.6_Pt_1.1422 2252262

[B27] Boskabady MH Kiani S Aslani MR Tracheal responsiveness to both isoprenaline and beta-adrenoreceptor blockade by propranolol in cigarette smoke exposed and sensitized guinea pigs Respirol 2006 11 572 578 10.1111/j.1440-1843.2005.00893.x 16916329

[B28] Canning BJ Chou Y Using guinea pigs in studies relevant to asthma and COPD Pulm Pharmacol Ther 2008 21 702 720 10.1016/j.pupt.2008.01.004 18462968 PMC2882537

[B29] Kasper G Mao L Geissler S Draycheva A Trippens J Kühnisch J Insights into mesenchymal stem cell aging: Involvement of antioxidant defense and actin cytoskeleton Stem cells 2009 27 1288 1297 10.1002/stem.49 19492299

[B30] Yun Luan XZ Kong F Cheng G-H Qi T-G Zhang Z-H Mesenchymal stem cell prevention of vascular remodeling in high flow-induced pulmonary hypertension through a paracrine mechanism Int Immunopharmacol 2012 14 432 437 10.1016/j.intimp.2012.08.001 22922316

[B31] Lee RH Pulin AA Seo MJ Kota DJ Ylostalo J Larson BL Intravenous hMSCs improve myocardial infarction in mice because cells embolized in lung are activated to secrete the anti-inflammatory protein TSG-6 Cell Stem Cell 2009 5 54 63 10.1016/j.stem.2009.05.003 19570514 PMC4154377

[B32] Furlani D Ugurlucan M Ong L Bieback K Pittermann E Westien I Is the intravascular administration of mesenchymal stem cells safe?: Mesenchymal stem cells and intravital microscopy Microvas Res 2009 77 370 376 10.1016/j.mvr.2009.02.001 19249320

[B33] Suckow MA Stevens KA Wilson RP The laboratory rabbit, guinea pig, hamster, and other rodents 2012 San Diego: Access Online via Elsevier

[B34] Boskabady MH Keyhanmanesh R Khamneh S Ebrahimi MA The effect of *Nigella sativa* extract on tracheal responsiveness and lung inflammation in ovalbuminsensitizedguinea pigs Clinics 2011 66 879 887 10.1590/S1807-59322011000500027 21789395 PMC3109390

[B35] Boskabady MH Bayrami G Tabatabaee A The effect of the extract of Crocus sativus and its constituent safranal, on lung pathology and lung inflammation of ovalbumin sensitized guinea-pigs Phyto Med 2012 19 904 911 10.1016/j.phymed.2012.05.006 22743244

[B36] Qamar W Sultana S Farnesol ameliorates massive inflammation, oxidative stress and lung injury induced by intratracheal instillation of cigarette smoke extract in rats: an initial step in lung chemoprevention Chem-BiolInteract 2008 176 79 87 10.1016/j.cbi.2008.08.011 18793622

[B37] Chen H Hansen MJ Jones JE Vlahos R Bozinovski S Anderson GP Cigarette smoke exposure reprograms the hypothalamic neuropeptide Y axis to promote weight loss Am J Resp Critic Care Med 2006 173 1248 1254 10.1164/rccm.200506-977OC 16531608

[B38] Kubo S Kobayashi M Masunaga Y Ishii H Hirano Y Takahashi K Cytokine and chemokine expression in cigarette smoke-induced lung injury in guinea pigs Eur Resp J 2005 26 993 1001 10.1183/09031936.05.00042405 16319327

